# The Effect of the APOE Genotype on Individual *BrainAGE* in Normal Aging, Mild Cognitive Impairment, and Alzheimer’s Disease

**DOI:** 10.1371/journal.pone.0157514

**Published:** 2016-07-13

**Authors:** Luise Christine Löwe, Christian Gaser, Katja Franke, for the Alzheimer’s Disease Neuroimaging Initiative

**Affiliations:** 1 Medical Faculty, University of Jena, Jena, Germany; 2 Structural Brain Mapping Group, Department of Neurology, University Hospital Jena, Jena, Germany; 3 Department of Psychiatry, University Hospital Jena, Jena, Germany; Nathan Kline Institute and New York University School of Medicine, UNITED STATES

## Abstract

In our aging society, diseases in the elderly come more and more into focus. An important issue in research is Mild Cognitive Impairment (MCI) and Alzheimer’s Disease (AD) with their causes, diagnosis, treatment, and disease prediction. We applied the Brain Age Gap Estimation (*BrainAGE*) method to examine the impact of the Apolipoprotein E (APOE) genotype on structural brain aging, utilizing longitudinal magnetic resonance image (MRI) data of 405 subjects from the Alzheimer’s Disease Neuroimaging Initiative (ADNI) database. We tested for differences in neuroanatomical aging between carrier and non-carrier of APOE ε4 within the diagnostic groups and for longitudinal changes in individual brain aging during about three years follow-up. We further examined whether a combination of *BrainAGE* and APOE status could improve prediction accuracy of conversion to AD in MCI patients. The influence of the APOE status on conversion from MCI to AD was analyzed within all allelic subgroups as well as for ε4 carriers and non-carriers. The *BrainAGE* scores differed significantly between normal controls, stable MCI (sMCI) and progressive MCI (pMCI) as well as AD patients. Differences in *BrainAGE* changing rates over time were observed for APOE ε4 carrier status as well as in the pMCI and AD groups. At baseline and during follow-up, *BrainAGE* scores correlated significantly with neuropsychological test scores in APOE ε4 carriers and non-carriers, especially in pMCI and AD patients. Prediction of conversion was most accurate using the *BrainAGE* score as compared to neuropsychological test scores, even when the patient’s APOE status was unknown. For assessing the individual risk of coming down with AD as well as predicting conversion from MCI to AD, the *BrainAGE* method proves to be a useful and accurate tool even if the information of the patient’s APOE status is missing.

## Introduction

During the last 20 years structural brain imaging was more and more integrated into research and diagnosis of neurological disorders [1]. It became part of the diagnostic workflow to assure clinical diagnosis, to clarify differential diagnoses [2] or to obtain longitudinal data for patient’s follow-up. Brain imaging is also increasingly used as diagnostic marker for abnormal brain atrophy processes such as in Alzheimer’s Disease (AD) [3–5]. AD is of great importance for research since it is the most common cause of dementia late in life, affecting approximately 1% of the population of 60–65 years, and 10–35% of 85 years and older [6].

Many AD patients suffer from Mild Cognitive Impairment (MCI) before fully developing all symptoms of AD. MCI is seen as prodromal state of AD [7] or transitional state between normal aging and AD [8]. In the case of cognitive impairment and dementia, the patterns and dimension of brain atrophy correlate strongly with the current and future extent of the disease [9–12]. Generally, whole brain atrophy rates are estimated to be about 1% per year in patients with very mild AD compared to about 0.5% in non-demented elderly [13], and approximately 2% per year for gray matter volume in AD patients [14].

During the last years, several methods have been developed to predict conversion from MCI to AD. Some of them are based on MR imaging, since it is easily applicable in clinics and widely available as well as non-invasive. MRI data can be also easily used for further analysis and calculations. Recently, a novel approach for estimating the individual neuroanatomical age based on structural MRI and a machine-learning pattern recognition method was presented, utilizing Relevance Vector Regression (RVR) to model brain aging in a large sample of healthy subjects [15]. Analyzing the local patterns of brain atrophy and matching them to the chronological age of the subject, a reliable biomarker based on the estimation of a person’s *brain age gap estimation (BrainAGE)* score was obtained. Applying the *BrainAGE* approach to clinical samples, this score discriminated those MCI subjects converting to AD within 36 months follow-up, i.e. progressive MCI (pMCI), from those remaining stable during 36 months follow-up, i.e. stable MCI (sMCI) [16]. Already at baseline MRI scan, pMCI and AD patients showed increased *BrainAGE* score of 6 to 7 years as compared to the control and sMCI groups. Additionally, brain aging was even accelerating by one year per follow-up year in the pMCI group and 1.5 years per follow-up year in the AD group during the follow-up period of about four years [17]. These findings are in line with other publications revealing dramatic shrinkage of brain tissue in MCI and AD [12, 13, 18, 19]. The scope of the present study was to investigate whether including individual apolipoprotein E (APOE) genotype status increases prediction accuracy for conversion from MCI to AD based on structural MRI.

The polymorphic APOE gene is located on chromosome 19q13.2 [20], with ε2, ε3, and ε4 being the three most common allelic isoforms [21, 22]. It is well known that APOE ε4 is a dose-dependent risk factor for developing late-onset AD [23–27]. Risk estimations vary between 3 times to a more than 4 times elevated risk per APOE ε4 allele [21, 23, 24]. Thus, AD affection would be around 8 to 15 times more likely in homozygous carriers of the APOE ε4 allele as compared to ε4 non-carriers [24, 26, 28]. APOE ε4 also influences the clinical course of AD [29–31], causing earlier onset of dementia [25–27, 32–35], higher degrees of brain atrophy [36], lower temporal [19, 37], hippocampal [2, 36–40], amygdala volumes [38, 41] and significant thinner cortices [42], and faster cognitive decline [30]. Most studies agree about the negative influence of APOE ε4 on disease severity of AD, manifesting in the deposit of neuritic plaques [43, 44] and neurofibrillary tangles [44]. In contrast, the APOE ε2-isoform is supposed to has a protective effect, e.g. manifesting in lower incidences of MCI and AD, older age of AD onset [21, 25, 27, 45, 46], and slower cognitive decline [30].

In the present study we analyzed the effects of the APOE status on individual deviations from normal brain aging trajectories, its longitudinal course as well as its relation to cognition and disease severity in healthy controls, MCI and AD patients. Additionally, we investigated whether a combination of *BrainAGE* and APOE status would increase prediction accuracy for conversion from MCI to AD.

## Methods

### Study samples

#### Longitudinal sample

Data used in the preparation of this article were obtained from the Alzheimer’s Disease Neuroimaging Initiative (ADNI) database (adni.loni.usc.edu). The ADNI was launched in 2003 as a public-private partnership, led by Principal Investigator Michael W. Weiner, MD. The primary goal of ADNI has been to test whether serial magnetic resonance imaging (MRI), positron emission tomography (PET), other biological markers, and clinical and neuropsychological assessment can be combined to measure the progression of mild cognitive impairment (MCI) and early Alzheimer’s disease (AD).

To investigate the longitudinal pattern of *BrainAGE* changes as a function of the APOE ε4 carrier status, this sample included all subjects from the ADNI database, for whom the APOE ε4 status as well as a baseline MRI scan and at least one follow-up MRI scan (1.5T) were available, resulting in a sample size of 405 subjects (Table 1). For the exact procedures of data collection and up-to-date information, see www.adni-info.org. Subjects were grouped as (i) NO (normal control group), if subjects were diagnosed cognitively healthy at baseline and remained so during 3 years follow-up (*n* = 107); (ii) sMCI (stable MCI), if subjects were diagnosed with MCI at baseline and remained so during 3 years follow-up (*n* = 36), (iii) pMCI (progressive MCI), if subjects were diagnosed with MCI at baseline and classified AD at some point during follow-up, without reversion to MCI or NO (*n* = 112), (iv) AD, if subjects were diagnosed with AD at baseline and remained so at any follow-up (*n* = 150).

**Table 1:** Characteristics of the longitudinal test sample.

	NO (*n* = 107)		sMCI (*n* = 36)		pMCI (*n* = 112)		AD (*n* = 150)		ANOVA (p)		
	ε4 carriers (ε2/ε4; ε3/ε4; ε4/ε4)	ε4 non carriers (ε2/ε3; ε3/ε3)	ε4 carriers (ε2/ε4; ε3/ε4; ε4/ε4)	ε4 non-carriers (ε2/ε3; ε3/ε3)	ε4 carriers (ε2/ε4; ε3/ε4; ε4/ε4)	ε4 non-carriers (ε2/ε3; ε3/ε3)	ε4 carriers (ε2/ε4; ε3/ε4; ε4/ε4)	ε4 non-carriers (ε2/ε3; ε3/ε3)	Diagnostic group	ε4 status (carriers vs. non-carriers)	diagnostic group x ε4 status
**No. of subjects (by APOE genotypes)**	26 (1 / 21 / 4)	81 (16 / 65)	14 (0 / 12 / 2)	22 (3 / 19)	78 (5 / 52 / 21)	34 (2 / 32)	101 (4 / 66 / 31)	49 (4 / 45)	-	-	-
**Baseline**											
**Age mean in years (SD)**	75.0 (5.1)	75.9 (4.9)	77.3 (5.6)	76.8 (6.5)	74.1 (6.5)	75.5 (9.3)	74.1 (6.8)	75.7 (8.9)	0.36	0.30	0.88
**MMSE mean (SD)**	29.3 (0.8)	29.2 (0.9)	27.7 (1.7)	27.2 (2.0)	26.7 (1.8)	26.4 (1.7)	23.4 (2.0)	23.5 (1.9)	**< 0.001**	0.34	0.71
**CDR-SB mean (SD)**	0.0 (0.0)	0.0 (0.1)	1.3 (0.6)	1.1 (0.6)	1.9 (1.0)	1.9 (1.1)	4.2 (1.5)	4.3 (1.7)	**< 0.001**	0.92	0.96
**ADAS mean (SD)**	8.3 (3.9)	8.9 (3.8)	17.3 (5.3)	17.3 (6.3)	21.8 (5.8)	21.8 (5.4)	28.7 (7.2)	29.0 (9.1)	**< 0.001**	0.79	0.99
***BrainAGE* score in years (SD)**	-0.11 (6.79)	-1.35 (6.45)	-0.88 (6.13)	0.09 (4.93)	5.83 (6.44)	5.54 (9.68)	5.76 (7.68)	6.20 (9.52)	**< 0.001**	0.97	0.85
**Last follow-up scan**											
**Follow-up duration in days (SD)**	1171 (234)	1197 (270)	1121 (283)	1110 (222)	967 (381)	974 (309)	616 (223)	595 (221)	**< 0.001**	0.99	0.94
**Age mean in years (SD)**	78.2 (5.1)	79.1 (5.0)	80.4 (5.4)	79.9 (6.5)	76.7 (6.7)	78.1 (9.4)	75.8 (6.9)	77.4 (9.1)	**< 0.05**	0.31	0.89
**MMSE mean (SD)**	28.5 (1.6)	29.2 (1.1)	26.7 (2.8)	27.4 (2.6)	21.4 (4.1)	21.9 (4.7)	19.2 (5.8)	19.2 (5.3)	**< 0.001**	0.31	**< 0.05**
**CDR-SB mean (SD)**	0.2 (0.5)	0.2 (0.5)	1.9 (1.0)	1.7 (1.2)	5.5 (2.6)	5.2 (2.5)	7.6 (3.7)	7.5 (3.7)	**< 0.001**	0.69	0.99
**ADAS mean (SD)**	10.0 (5.7)	10.2 (5.4)	18.0 (7.1)	17.4 (6.2)	32.1 (8.0)	33.8 (12.2)	38.9 (12.2)	36.6 (12.1)	**< 0.001**	0.84	0.46
***BrainAGE* score in years (SD)**	-0.16 (7.94)	-1.40 (6.06)	-0.01 (6.05)	-0.64 (4.77)	8.68 (7.24)	7.34 (10.29)	8.30 (8.03)	7.67 (10.14)	**< 0.001**	0.32	0.98
**Changing rates (per follow-up year)**											
**MMSE**	-0.17	-0.01	-0.26	0.10	-2.20	-1.83	-2.42	-2.47	**< 0.001**	0.38	0.87
**CDR-SB**	0.06	0.03	0.19	0.24	1.40	1.32	1.81	1.82	**< 0.001**	0.92	0.99
**ADAS**	0.51	-0.04	-0.11	-0.06	3.80	4.31	5.62	4.16	**< 0.001**	0.42	0.26
***BrainAGE***	-0.01	0.03	0.20	-0.13	1.13	0.61	1.68	0.90	**< 0.001**	**< 0.05**	0.25

*P*-values are resulting from ANOVA. **Bold** type = significant test results.

The following neuropsychiatric scales, administered at baseline and follow-up examinations, were used to evaluate the degree of cognitive decline: Alzheimer’s Disease Assessment Scale (ADAS; ranging from 0 to 85, with higher test scores indicating worse cognitive functioning) [47], global Clinical Dementia Rating Scale Sum of Boxes (CDR; ranging from 0 to 3, with 0 indicating NO, 0.5 denoting MCI, 1 and more indicates stages of AD) [48], and Mini-Mental State Examination (MMSE; ranging from 0 to 30, with lower test scores indicating worse cognitive functioning) [49].

#### Sample for prediction of AD conversion

To explore the performance of the *BrainAGE* framework in predicting conversion from MCI to AD in APOE ε4 carriers and non-carriers, all MCI subjects were included for whom baseline MRI data (1.5T), at least moderately confident diagnoses (i.e. confidence >2), and test scores in certain cognitive scales (i.e., ADAS, CDR-SB, MMSE) were available. The MCI subjects (*n* = 193) were grouped as (i) *sMCI* (stable MCI), if diagnosis was MCI stable during follow-up, at least for 36 months (*n* = 62); (ii) *pMCI_early* (progressive MCI), if diagnosis was MCI at baseline measurement and conversion to AD occurred within the first 12 months after baseline, without reversion to MCI or cognitive normal (NO) at any follow-up (*n* = 57); (iii) *pMCI_late*, if diagnosis was MCI at baseline measurement and conversion to AD was diagnosed after the first 12 months (i.e. at 18, 24, or 36 months follow-up), without reversion to MCI or NO at any follow-up (*n* = 74). Hereby, time to conversion does refer to time from being enrolled in ADNI (i.e., individual baseline measurements) till first diagnose of AD. Details of the characteristics of the prediction sample are presented in Table 2. The participants were further grouped according to their APOE ε4 status, resulting in ε4 carrier groups (sMCI^C^, pMCI^C^_early, pMCI^C^_late) and non-carrier groups (sMCI^NC^, pMCI^NC^_early, pMCI^NC^_late).

**Table 2:** Baseline characteristics of the MCI sample used for prediction of AD conversion.

	ε4 carriers (*n* = 117)			ε4 non-carriers (*n* = 76)			ANOVA (*p*)		
	sMCI^C^	pMCI^C^_ early	pMCI^C^_ late	sMCI^NC^	pMCI^NC^_ early	pMCI^NC^_ late	Diagnostic group	ε4 status	Group x ε4 status
**No. subjects**	26	33	58	36	24	16	-	-	-
**Males / Females**	23 / 3	20 / 13	36 / 22	26 / 10	13 / 11	11 / 5	-	-	-
**Age mean (SD)**	76.5 (5.2)	72.9 (6.0)	75.0 (6.4)	76.2 (6.8)	75.3 (8.3)	76.4 (10.0)	0.20	0.26	0.55
**Education years mean (SD)**	16.3 (2.7)	15.7 (2.6)	15.9 (3.0)	16.6 (2.5)	15.0 (3.4)	16.1 (2.6)	0.12	0.97	0.61
**MMSE mean (SD)**	28.0 (1.4)	26.5 (2.0)	26.8 (1.5)	27.5 (2.0)	26.4 (1.8)	26.6 (1.7)	**< 0.001**	0.33	0.72
**CDR-SB mean (SD)**	1.4 (0.7)	2.1 (0.9)	1.7 (0.9)	1.3 (0.6)	1.9 (0.9)	2.0 (1.1)	**< 0.001**	0.87	0.42
**ADAS mean (SD)**	17.1 (5.2)	23.7 (6.6)	20.6 (4.4)	15.7 (6.1)	23.1 (5.9)	19.7 (4.2)	**< 0.001**	0.24	0.93
***BrainAGE* mean (SD)**	0.0 (4.4)	9.0 (6.3)	5.7 (6.0)	1.2 (4.0)	8.0 (9.2)	5.0 (7.7)	**< 0.001**	0.42	0.38

*P*-values are resulting from ANOVA. **Bold** type = significant test results.

### MRI Data Preprocessing and Data Reduction

Preprocessing of the T1-weighted images was done using the SPM8 package (http://www.fil.ion.ucl.ac.uk/spm) and the VBM8 toolbox (http://dbm.neuro.uni-jena.de), running under Matlab. All T1-weighted images were corrected for bias-field inhomogeneities, then spatially normalized and segmented into gray matter, white matter, and cerebrospinal fluid within the same generative model [50]. The segmentation procedure was further extended by accounting for partial volume effects [51], by applying adaptive maximum a posteriori estimations [52], and by using a hidden Markov random field model [53]. Preprocessing the images further included affine registration and smoothing with 4-mm full-width-at-half-maximum (FWHM) smoothing kernels. Spatial resolution was set to 4 mm. Data reduction was performed by applying Principal Component Analysis (PCA) utilizing the “Matlab Toolbox for Dimensionality Reduction” (http://ict.ewi.tudelft.nl/~lvandermaaten/Home.html). PCA was performed on the training sample only. The estimated transformation parameters were subsequently applied to the test samples. No further data reduction or region pre-selection was accomplished.

### Estimation of *BrainAGE* scores

**Fig 1:**
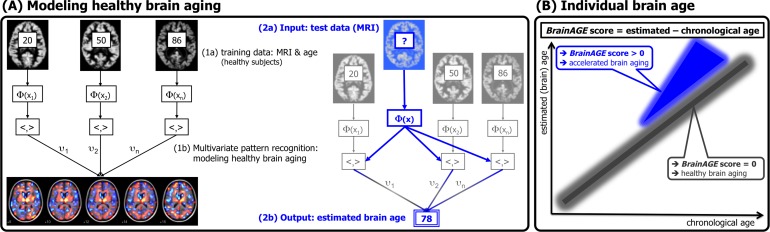
Depiction of the *BrainAGE* concept. (A) The model of healthy brain aging is trained with the chronological age and preprocessed structural MRI data of a training sample (left, with an exemplary illustration of the most important voxel locations that were used by the age regression model). Subsequently, the individual brain ages of previously unseen test subjects are estimated, based on their MRI data (blue, picture modified from [56]). (B): The difference between the estimated and chronological age results in the *BrainAGE* score, indicating abnormal brain aging. [Image reproduced from [17], with permission from Hogrefe Publishing, Bern]

The *BrainAGE* framework utilizes a machine-learning pattern recognition method, namely relevance vector regression (RVR) [54], to estimate individual brain ages based on T1-weighted MR images [15]. The brain age of each test subject can be estimated using the individual tissue-classified MRI data, aggregating the complex, multidimensional aging pattern across the whole brain into one single value (Fig 1A). The difference between estimated and true chronological age will reveal the individual *Brain Age Gap Estimation* (*BrainAGE*) score. Consequently, the *BrainAGE* score directly quantifies the amount of acceleration or deceleration in brain aging. For example, if a 70 years old individual has a *BrainAGE* score of +5 years, this means that this individual shows the typical atrophy pattern of a 75 year old individual (Fig 1B). Recent work has demonstrated that this method provides reliable and stable estimates, with a correlation of *r =* 0.92 between the estimated and the chronological age and a mean absolute error of 5 years in healthy subjects aged 20–86 years [15]. Additionally, *BrainAGE* scores calculated from two shortly delayed scans resulted in an intraclass correlation coefficient (ICC) of 0.93 [55].

For the present study, the *BrainAGE* method was applied using the preprocessed gray matter images (as described above). To train the age estimation framework, we used T1-weighted MRI data of all subjects from the publicly accessible database “Information eXtraction from Images” (IXI; http://www.brain-development.org; data downloaded in September 2011) aged 20–86 years (mean age 48.6 ± 16.5 years; *n* = 560), which were collected on three different scanners (Philips 1.5T, General Electric 1.5T, Philips 3.0T). Additionally, MRI data of all healthy control subjects from the publicly accessible database “Open Access Series of Imaging Studies” (OASIS; http://www.oasis-brains.org; downloaded in June 2009) aged 51–94 years (mean age 71.3 ± 11.8 years; *n* = 126) were also included in the training sample. For training the model as well as for predicting individual brain ages, we used “The Spider” (http://www.kyb.mpg.de/bs/people/spider/main.html), a freely available toolbox running under Matlab. For an illustration of the most important features (i.e., the importance of voxel locations for regression with age) that were used by the RVR to model normal brain aging and more detailed information please refer to [15]. In both test samples, *BrainAGE* scores were calculated based on baseline MRI. In the longitudinal test sample, follow-up *BrainAGE* scores were calculated based on each available MRI data during follow-up.

### Statistical Analysis

First, baseline *BrainAGE* scores, *BrainAGE* scores at last visit, and longitudinal changes in *BrainAGE* were compared among the 4 diagnostic groups and the APOE ε4 carrier status using analysis of variance (ANOVA). Post-hoc analyses (with Bonferroni correction to compensate for multiple comparisons) were conducted to further explore group differences. Additionally, the effects of the particular allelic isoforms (i.e., ε2/ε3, ε3/ε3, ε2/ε4, ε3/ε4, ε4/ε4) on *BrainAGE* were analyzed. Longitudinal changes in individual *BrainAGE* scores, i.e., the differences between follow-up and baseline *BrainAGE* scores were fitted against days from baseline with a multivariate linear regression model, including correction for age and gender. The relationships between *BrainAGE* scores and cognitive scales (i.e. MMSE, CDR-SB, ADAS) were explored using Pearson’s linear correlation coefficients.

In the second part of the study, prediction of conversion from MCI to AD in APOE ε4 carriers and non-carriers based on baseline *BrainAGE* scores was studied. Receiver operating characteristics (ROC) for discriminating MCI subjects who converted to AD from those who remained stable during follow-up were computed in early converting as well as all MCI subjects together, resulting in the area under the curve (AUC), also known as C-statistics or c-index. The AUC shows the quality of classification, with 1.0 indicating a perfect discrimination and 0.5 indicating a result obtained by chance only. In order to test whether the resulting AUC derived from ROC analysis based on *BrainAGE* scores is statistically greater than the AUCs of the cognitive scores, one-tailed z-tests were performed. Additionally, the McNemar test for paired data was performed in order to statistically test whether predictions of conversion based on baseline *BrainAGE* scores are significantly better than predictions based on cognitive scores. Furthermore, univariate Cox regression was used to estimate the hazard rate for conversion to AD, adjusted for age, gender, and education years. The time-to-event variable was time from baseline visit to first visit with AD diagnosis for pMCI subjects. For sMCI subjects, the duration of follow-up was truncated at 36 months. The main predictor was the baseline *BrainAGE* score as a continuous variable initially and with median split subsequently. Cox regression was also performed with baseline cognitive scores as main predictors. Furthermore, it was tested whether including the individual APOE status into the Cox regression model would significantly improve the model performance. As checked by log-minus-log-plots of survival, the assumption of proportional hazards was met for all Cox proportional hazard models. Cox regression was performed using SPSS. All other statistical testing was performed using Matlab.

## Results

### Longitudinal sample

*BrainAGE* scores and cognitive tests at baseline and follow-up were analyzed in all diagnostic groups (NO, sMCI, pMCI, AD) according to APOE ε4 carrier status (Table 1) and particular allelic isoform (Table 3). The allelic combination of ε2/ε2 was not represented in this sample. In line with other studies [22, 29], APOE ε2/ε4 was assigned to the ε4 carrier group.

**Table 3:** Mean BrainAGE scores at baseline and last follow-up for all particular allelic isoforms within the diagnostic groups of the longitudinal sample.

	NO		sMCI		pMCI		AD	
	Baseline	Last follow-up	Baseline	Last follow-up	Baseline	Last follow-up	Baseline	Last follow-up
**APOE ε2 / ε3**								
**No. subjects**	16		3		2		4	
**BrainAGE mean (SD)**	-2.66 (5.32)	-3.01 (5.42)	+1.95 (6.92)	+2.71 (5.45)	+3.43 (5.29)	+9.24 (2.10)	+8.80 (4.86)	+11.31 (6.16)
**APOE ε3 / ε3**								
**No. subjects**	65		19		32		45	
**BrainAGE mean (SD)**	-1.03 (6.69)	-1.01 (6.18)	-0.21 (4.73)	-1.16 (4.60)	+5.67 9.93)	+7.22 (10.60)	+5.97 (9.82)	+7.35 (10.40)
**APOE ε2 / ε4**								
**No. subjects**	1		0		5		4	
**BrainAGE mean (SD)**	+13.28 (0.00)	+11.72 (0.00)	-	-	+3.39 (6.72)	+7.25 (6.05)	+2.10 (10.60)	+3.29 (7.97)
**APOE ε3 / ε4**								
**No. subjects**	21		12		52		66	
**BrainAGE mean (SD)**	-1.42 (6.44)	-1.28 (8.07)	-0.15 (6.28)	+0.47 (6.45)	+5.38 (5.85)	+7.40 (7.03)	+6.19 (8.74)	+8.45 (8.60)
**APOE ε4 / ε4**								
**No. subjects**	4		2		21		31	
**BrainAGE mean (SD)**	+3.44 (4.44)	+2.75 (4.94)	-5.29 (2.93)	-2.85 (0.59)	+7.52 (7.64)	+12.18 (7.12)	+5.33 (5.18)	+8.63 (6.69)

**Fig 2:**
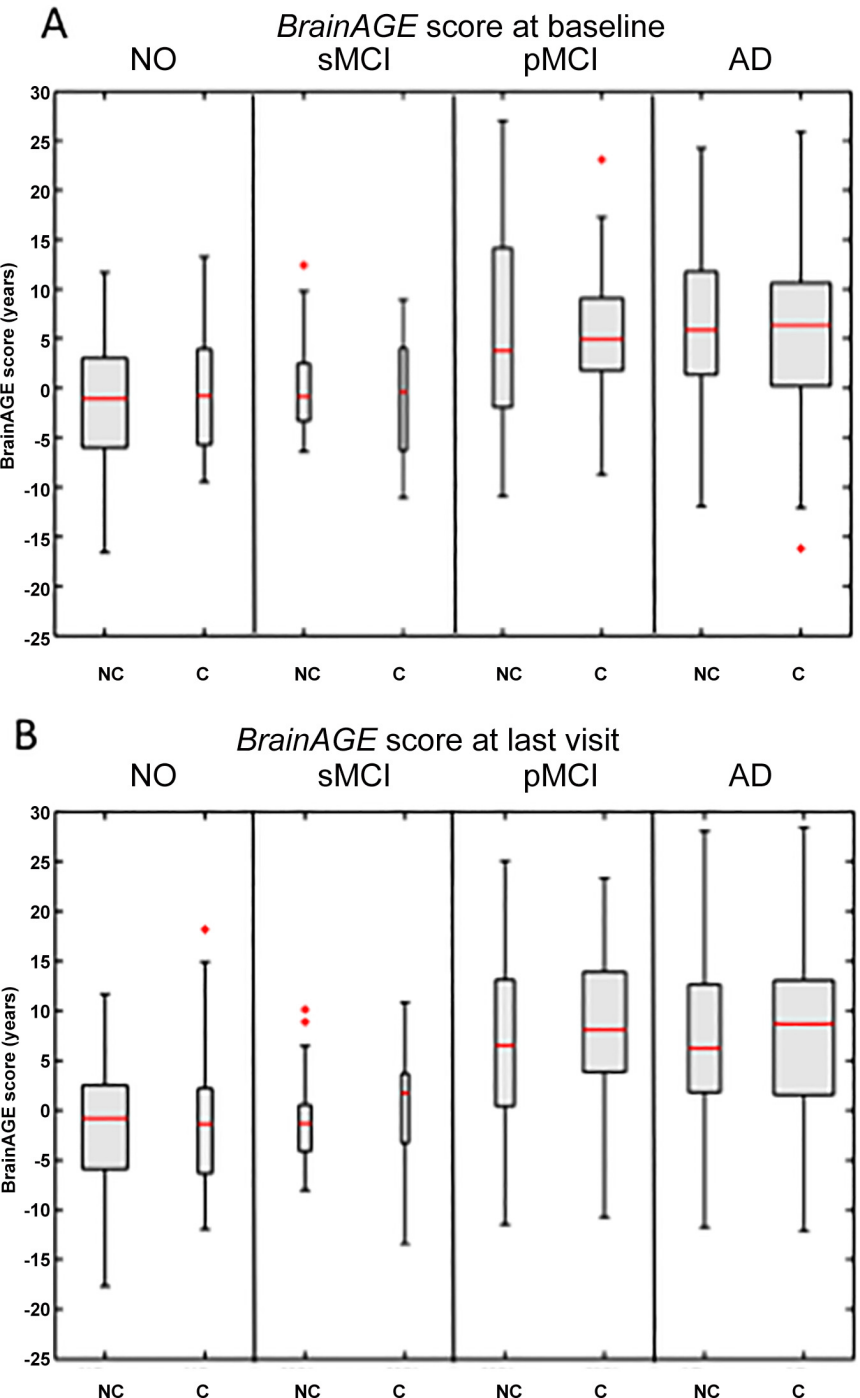
***BrainAGE* scores at (A) baseline and (B) the last visit for non-carriers and carriers of APOE ε4.** Shown are boxplots, presenting the distribution of the *BrainAGE* scores for the 4 diagnostic groups NO, sMCI, pMCI and AD. *BrainAGE* scores differed significantly between diagnostic groups at baseline (*F =* 18.9, *p <* 0.001) and at follow-up scans (*F =* 30.6, *p <* 0.001). Post-hoc tests showed significant differences between *BrainAGE* scores in NO as well as sMCI from BrainAGE scores in pMCI as well as AD at baseline and last visit (*p <* 0.05). The boxes include values between the 25^th^ and 75^th^ percentiles and the median (red line). Lines extending the boxes below and above include data within 1.5 times the interquartile range. All outliers are symbolized with a red”+”. Width of the boxes symbolizes group size.

*BrainAGE* scores differed significantly among all 4 diagnostic groups at baseline (*F* = 18.86, *p* < 0.001; Table 1) and at last MRI scan (*F* = 30.56, *p* < 0.001; Table 1). As revealed by post-hoc t-tests, *BrainAGE* scores in NO as well as sMCI differed significantly from *BrainAGE* scores in pMCI as well as AD at baseline (*p* < 0.05; Fig 2A) and at last MRI scan (*p* < 0.05; Fig 2B), suggesting neuroanatomical changes that show patterns of advanced brain aging in pMCI and AD patients. At baseline as well as at last MRI scan, there was no significant effect regarding APOE ε4 status or interaction between diagnostic group and APOE ε4 status. Additionally, no significant effects were found for the particular allelic isoforms (Table 3), which may be due to the very small number of patients for some allelic isoforms.

As mentioned above, patients with the allelic isoform ε2/ε4 were assorted to carriers. Since there were no representatives of this isoform in the sMCI cohort, we subsequently examined the possible effect of falsification by excluding all patients with a combination of ε2/ε4 from our longitudinal sample. However, test results did not change (F-statistics at baseline for diagnostic group: *F =* 9.22, *p <* 0.001; APOE ε4 status: *F =* 0.01, *p =* 0.99; interaction: *F =* 0.6, *p =* 0.79; follow-up scan for diagnostic group: *F =* 16.35, *p <* 0.001; APOE ε4 status: *F =* 0.62, *p =* 0.60; interaction: *F =* 0.74, *p =* 0.67).

**Fig 3:**
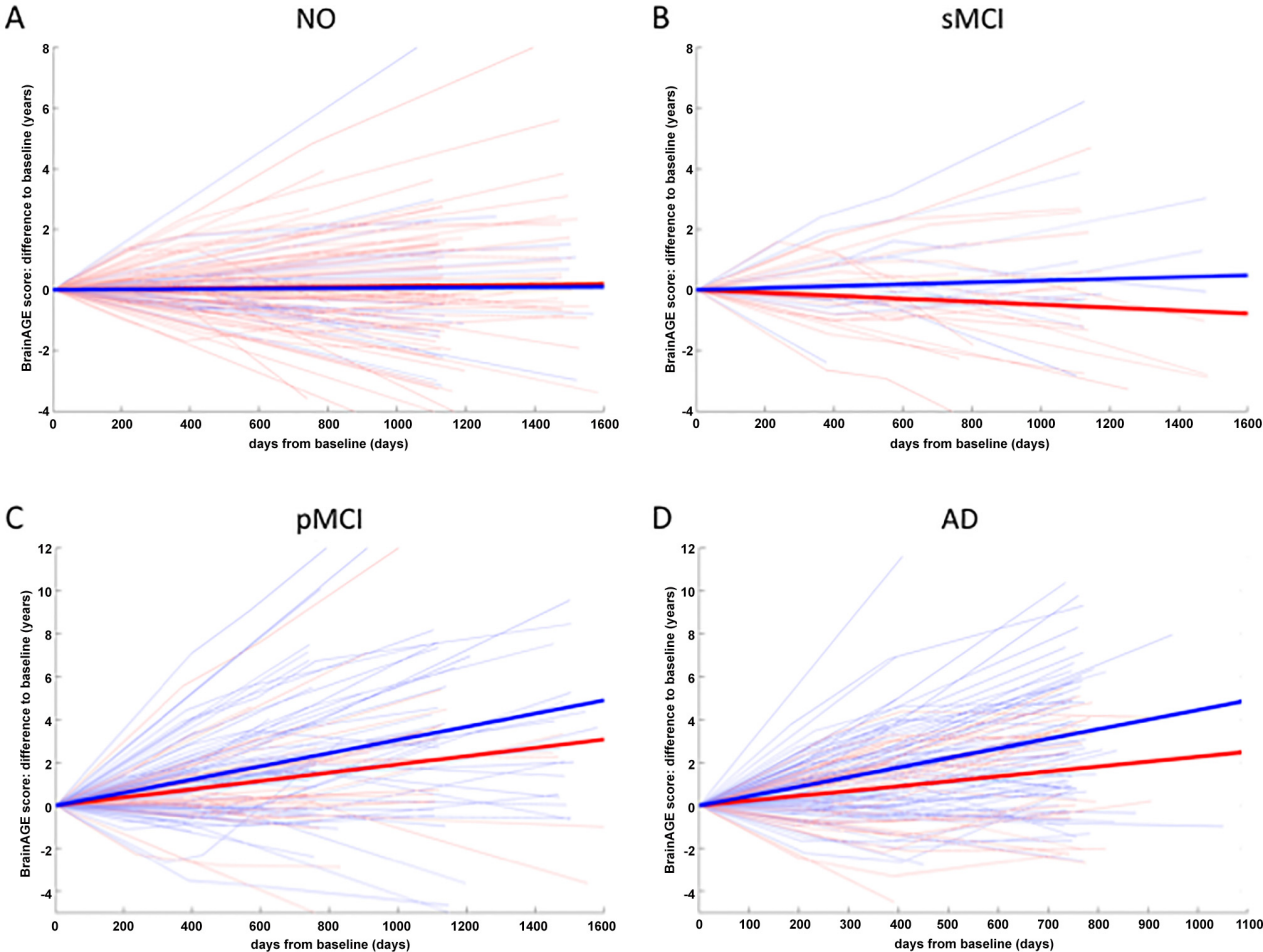
**Longitudinal changes in *BrainAGE* score for (A) NO, (B) sMCI, (C) pMCI and (D) AD patients.** Thin lines represent individual trajectories of *BrainAGE* score over time of follow-up. Thick lines represent the estimated average regression lines for APOE ε4 non-carriers (red) and carriers (blue).

**Fig 4:**
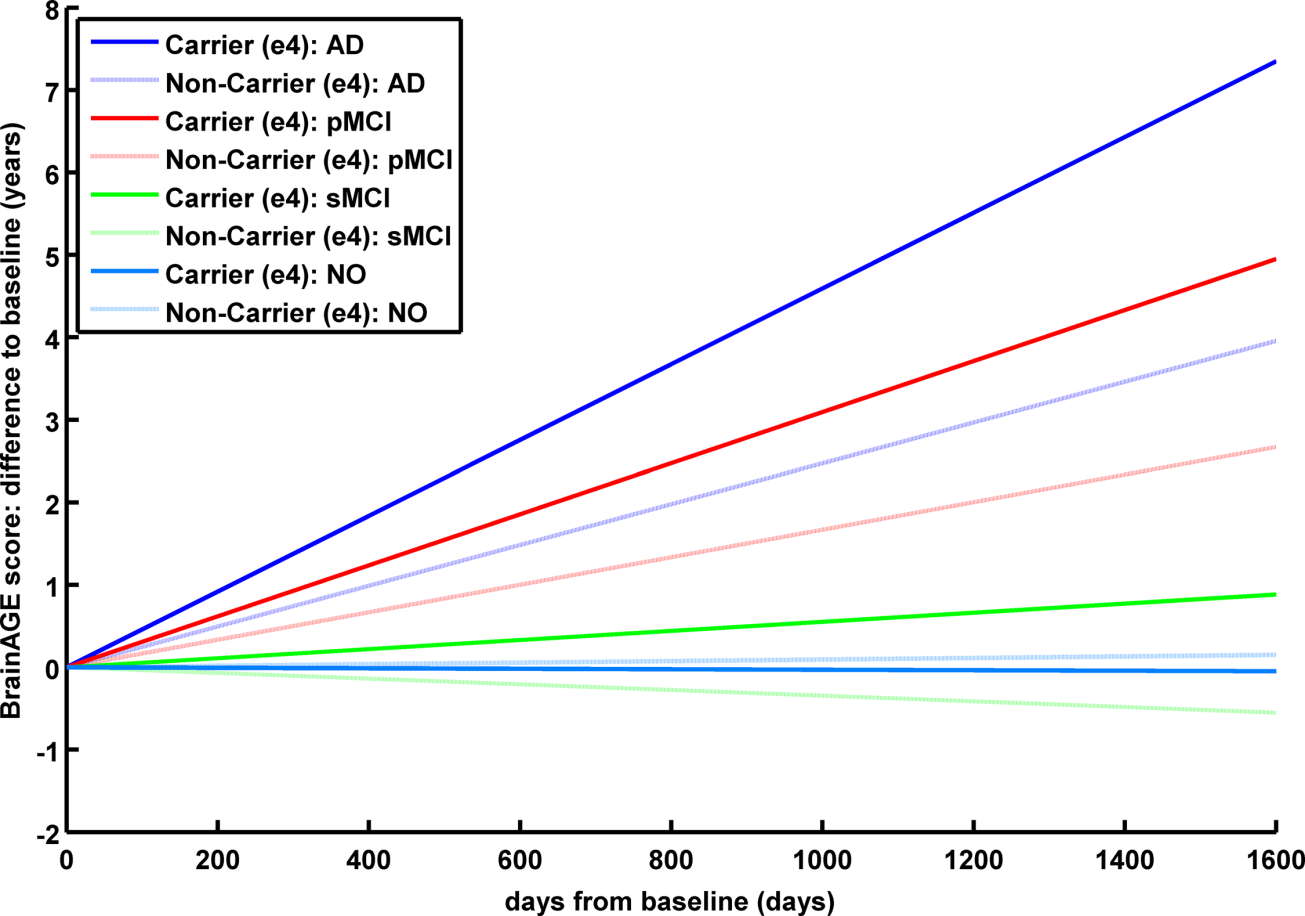
**Estimated longitudinal changes in *BrainAGE* scores for the 4 diagnostic groups:** NO (light blue), sMCI (green), pMCI (red) and AD (blue), subdivided into APOE ε4 carriers and non-carriers. Post-hoc t-tests resulted in significant differences for ε4 carriers and non-carriers as well as for NO / sMCI vs. pMCI / AD (*p <* 0.05).

To further investigate individual trajectories of *BrainAGE* scores, longitudinal changes as compared to the baseline assessment were analyzed for each available time point during follow-up. *BrainAGE* scores remained stable in the NO and sMCI groups across the follow-up period of about three years, but increased in the pMCI and AD groups, suggesting additional acceleration in brain aging in the pMCI and AD groups (Fig 3). *BrainAGE* changing rates differed significantly between NO and sMCI subjects as compared to pMCI and AD subjects as well as between APOE ε4 carriers and non-carriers (*p <* 0.05; Fig 4), with ε4 carriers showing increased changing rates as compared to non-carriers (Table 1).

Correlations between *BrainAGE* and cognitive scores were analyzed for baseline and last follow-up visit. In the whole sample, *BrainAGE* scores correlated significantly with each of the cognitive scores independent of the APOE ε4 carrier status (Table 4). Analyzing the diagnostic groups separately, correlations between *BrainAGE* and cognitive scores were only found in pMCI and AD patients, for both ε4 carriers and non-carriers. Probably, disease related neuroanatomical alterations might be reflected in the cognitive scores if they exceed a certain degree. Distinct differences between ε4 carriers and non-carriers were not found (Table 4).

**Table 4:** Correlation coefficients between *BrainAGE* scores and cognitive functioning (ADAS scores) as well as disease severity (MMSE & CDR-SB scores) for each diagnostic group and the whole test sample, separately for APOE ε4 carriers and non-carriers.

	NO		sMCI		pMCI		AD		*Whole sample*	
	ε4 carriers	ε4 non-carriers	ε4 carriers	ε4 non-carriers	ε4 carriers	ε4 non-carriers	ε4 carriers	ε4 non-carriers	*ε4 carriers*	*ε4 non-carriers*
No. of subjects	26	81	14	22	78	34	101	49	***219***	***186***
**Correlation with *BrainAGE* score at** **baseline**										
MMSE score	0.04	-0.21	0.45	-0.08	-0.08	-0.29	-0.28**	-0.62***	*-0*.34***	*-0*.52***
CDR-SB score	-0.04	-0.15	-0.12	0.02	0.27*	0.13	0.10	0.60***	*0*.29***	*0*.50***
ADAS score	-0.06	0.04	-0.45	-0.07	0.27*	0.32	0.19	0.52***	*0*.33***	*0*.50***
**Correlation with *BrainAGE* score at** **last scan**										
MMSE score	0.00	-0.17	0.30	-0.12	-0.21	-0.33	-0.38***	-0.66***	*-0*.44***	*-0*.59***
CDR-SB score	-0.07	0.00	-0.07	0.09	0.38**	0.23	0.21*	0.59***	*0*.40***	*0*.57***
ADAS score	-0.06	0.04	-0.34	0.09	0.38**	0.38*	0.25*	0.56***	*0*.43***	*0*.58***

***p<0.001

**p<0.01

*p<0.05.

### Prediction of conversion to AD

The subsample used to predict conversion to AD consisted of 193 MCI patients, including 117 APOE ε4 carriers and 76 non-carriers. Chronological age and education years did not differ between stable, early converting, and late converting MCI patients. Baseline *BrainAGE* scores differed significantly between groups (*F =* 8.96, *p <* 0.001; Table 2), as did the cognitive scores. There weren’t any effects for the APOE ε4 status or for interactions between diagnostic group and APOE ε4 status (Table 2).

A total number of 91 ε4 carriers and 40 non-carriers converted to AD during the 36 months of follow-up. That corresponds to a pre-test probability of 78% in carriers and 53% in non-carriers. In ε4 carriers, 28% of the MCI subjects converted to AD within the first 12 months after baseline examination, whereas 50% converted to AD after the first year of follow-up. In non-carriers, 32% of the MCI subjects converted to AD within the first 12 months after baseline examination, whereas 21% converted to AD after the first year of follow-up.

**Fig 5:**
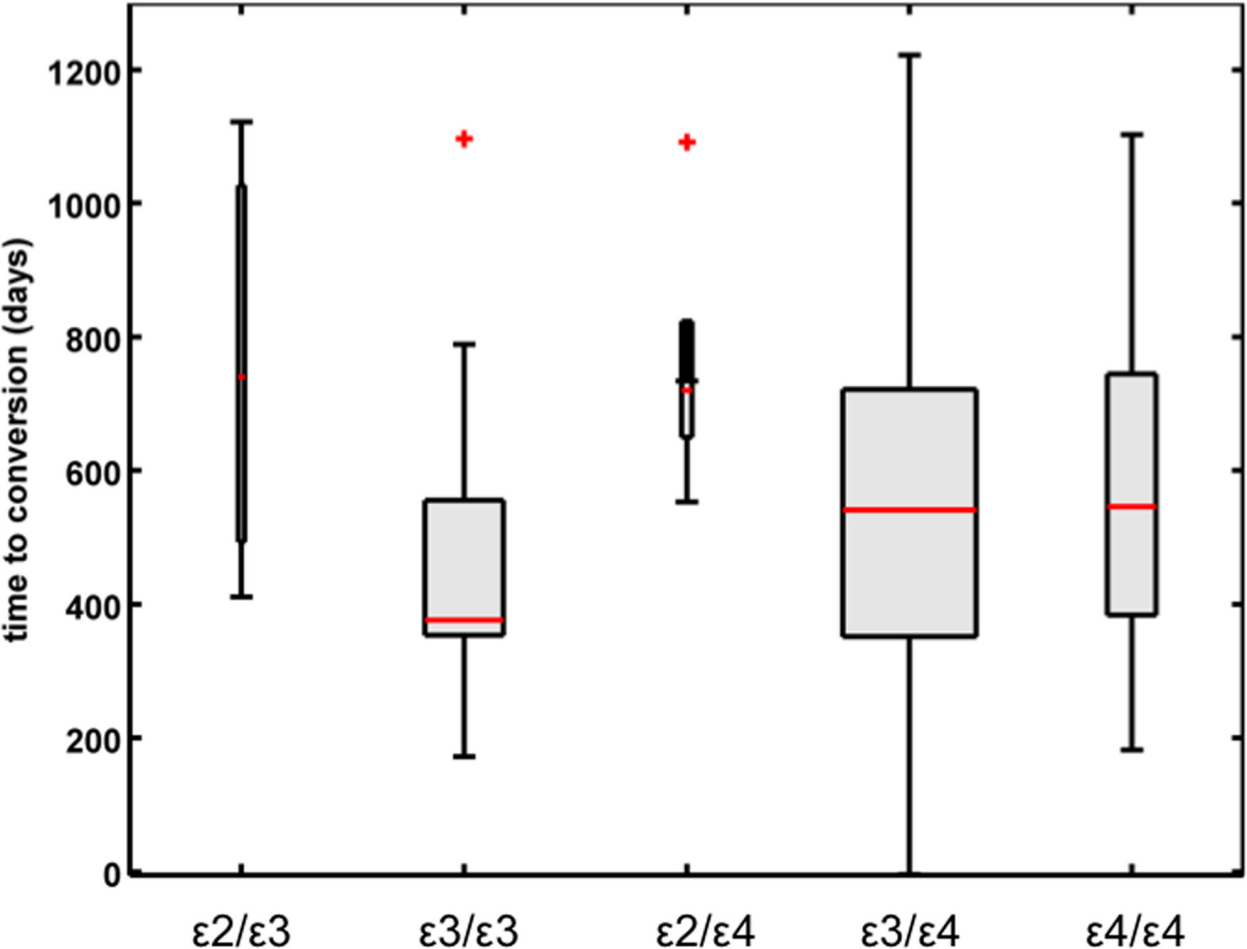
Mean days to conversion from MCI to AD subdivided into all allelic combinations of APOE. Presented are the mean ± SD days to conversion within the given allelic combinations of APOE (*F =* 3.14; *p =* 0.08): ε2/ε3 (*n =* 2): 758 ± 356, ε3/ε3 (*n =* 32): 448 ± 210, ε2/ε4 (*n =* 5): 756 ± 201, ε3/ε4 (*n =* 52): 534 ± 292, ε4/ε4 (*n =* 21): 591 ± 246. The boxes include values between the 25^th^ and 75^th^ percentiles and the median (red line). Lines extending the boxes below and above include data within 1.5 times the interquartile range. All outliers are symbolized with a red”+”. Width of the boxes symbolizes group size.

Regarding time to conversion from MCI to AD diagnosis, APOE ε4 carriers showed the tendency to take about 3 months longer to convert to AD (560 ± 280 days) as compared to non-carriers (471 ± 233 days; *F =* 3.14; *p =* 0.08; Fig 5). Interestingly, time to conversion was longer in homozygous ε4 carriers (591 days) as compared to homozygous ε3 carriers (448 days). Longest time to conversion was shown in heterozygous carriers of a protective ε2 allele with either ε3 (758 days) or ε4 (756 days). Time to conversion did not cover the whole time suffering from MCI, but rather corresponded to the individual time being enrolled in ADNI while suffering from MCI until AD was diagnosed for the first time.

**Fig 6:**
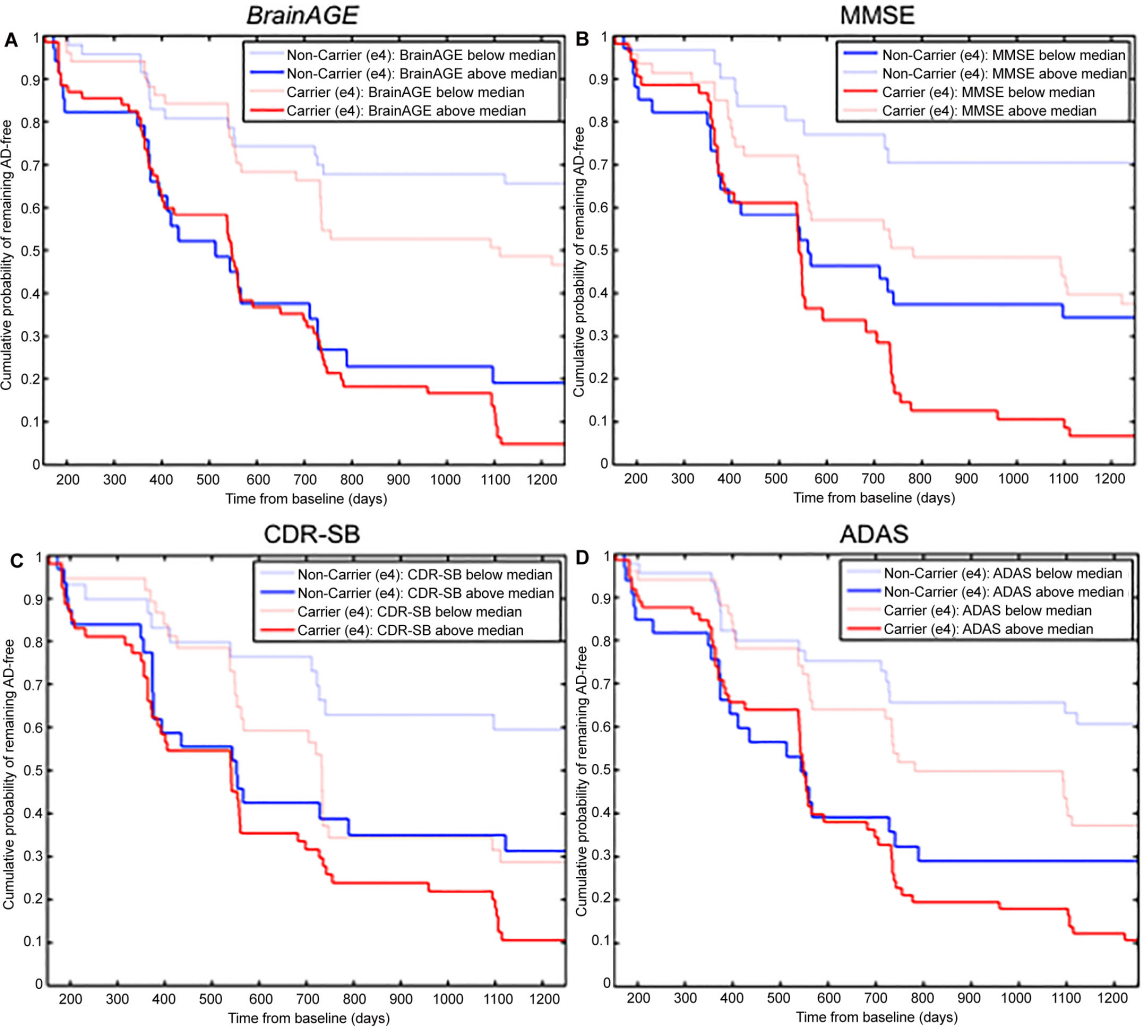
**Cumulative probability for MCI patients of remaining AD-free**, divided into patients with the score of interest below the median (light lines) and above it (dark lines). Non-carriers of the APOE ε4 gene are painted in blue, carriers in red. Shown are Kaplan-Meier survival curves based on Cox regression, comparing the cumulative incidence of AD in ε4 carriers and non-carriers in (A) *BrainAGE*, (B) MMSE, (C) CDR-SB, and (D) ADAS scores. The follow-up duration is truncated at 1250 days.

Cox regression for prediction of conversion to AD based on baseline *BrainAGE* scores resulted in higher baseline *BrainAGE* scores being associated with a higher risk of converting to AD independent of APOE status (*χ*^*2*^ *=* 53.88, *p <* 0.001; Table 5). Subjects with a *BrainAGE* score above median of 4.5 years had a nearly 4 times greater risk of converting to AD as compared to subjects with *BrainAGE* scores below the median (hazard ratio; HR: 3.76, *p <* 0.001; Table 5). Including the APOE status into the Cox regression model, the quality of the prediction model tended to improve (*χ*^*2*^ *=* 3.23, *p =* 0.07). The Cox regression model based on baseline *BrainAGE* scores outperformed all models based on baseline MMSE, CDR-SB, and ADAS scores, even when including the APOE ε4 status into the models (Table 5, Fig 6).

**Table 5:** Cox Regression values for cumulative AD incidence in APOE ε4 carriers and non-carriers in the *BrainAGE*, MMSE, CDR-SB, and ADAS scores alone and in combination with the APOE ε4 carrier status, based on a median split.

	Test of model		Change from model without APOE		Hazard ratio (HR)	Confidence interval (CI)	*p*
	*χ*^*2*^	*p*	*χ*^*2*^	*p*			
***BrainAGE***							
*BrainAGE* (only)*	53.88	**< 0.001**			3.76	2.58–5.48	**< 0.001**
***BrainAGE* & APOE**	56.79	**< 0.001**	3.23	0.07			
*° BrainAGE*					3.58	2.44–5.24	**< 0.001**
° APOE					1.41	0.96–2.05	0.08
**MMSE**							
**MMSE (only)**	18.46	**< 0.001**			2.37	1.58–3.55	**< 0.001**
**MMSE & APOE**	27.68	**< 0.001**	9.62	**< 0.01**			
° MMSE					2.42	1.61–3.63	**< 0.001**
° APOE					1.91	1.25–2.93	**< 0.01**
**CDR-SB**							
**CDR-SB (only)**	12.91	**< 0.001**			2.05	1.37–3.05	**< 0.001**
**CDR-SB & APOE**	19.61	**< 0.001**	6.95	**< 0.01**			
° CDR-SB					1.97	1.32–2.93	**< 0.001**
° APOE					1.72	1.14–2.60	**< 0.01**
**ADAS**							
**ADAS (only)**	22.57	**< 0.001**			2.35	1.63–3.38	**< 0.001**
**ADAS & APOE**	26.62	**< 0.001**	4.27	**< 0.05**			
° ADAS					2.22	1.54–3.20	**< 0.001**
° APOE					1.48	1.01–2.18	**< 0.05**

**Bold type** = significant; **asterisk** = best performance of all models.

**Fig 7:**
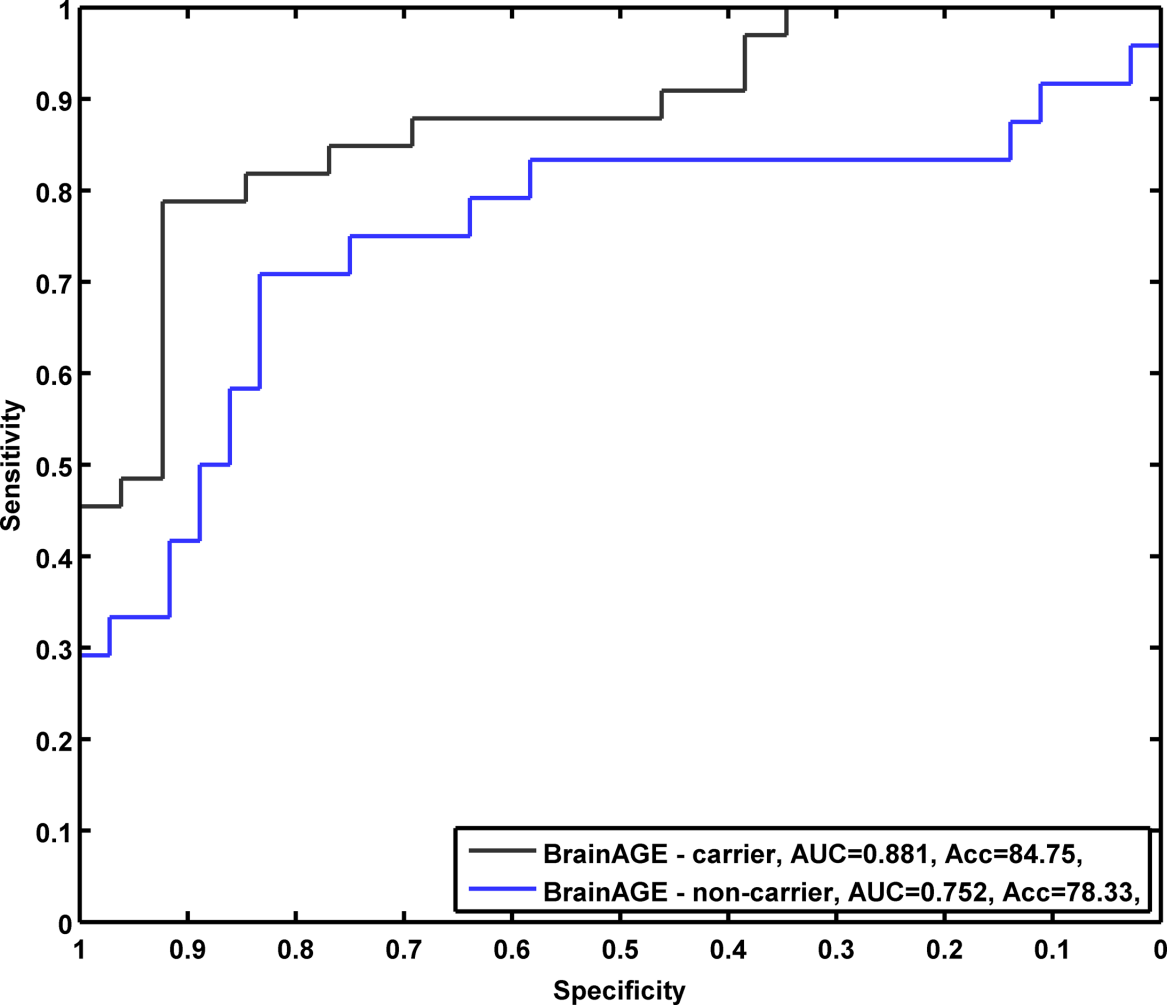
**ROC curves for pMCI_early subjects**, analyzing individual subject classification based on baseline *BrainAGE* score in either remaining MCI or converting to AD, subdivided into APOE ε4 carriers and non-carriers. Achieved accuracies (sensitivity / specificity) for predicting conversion from MCI to AD for APOE ε4 carriers: 85% (0.79 / 0.92), non-carriers: 78% (0.71 / 0.83).

**Fig 8:**
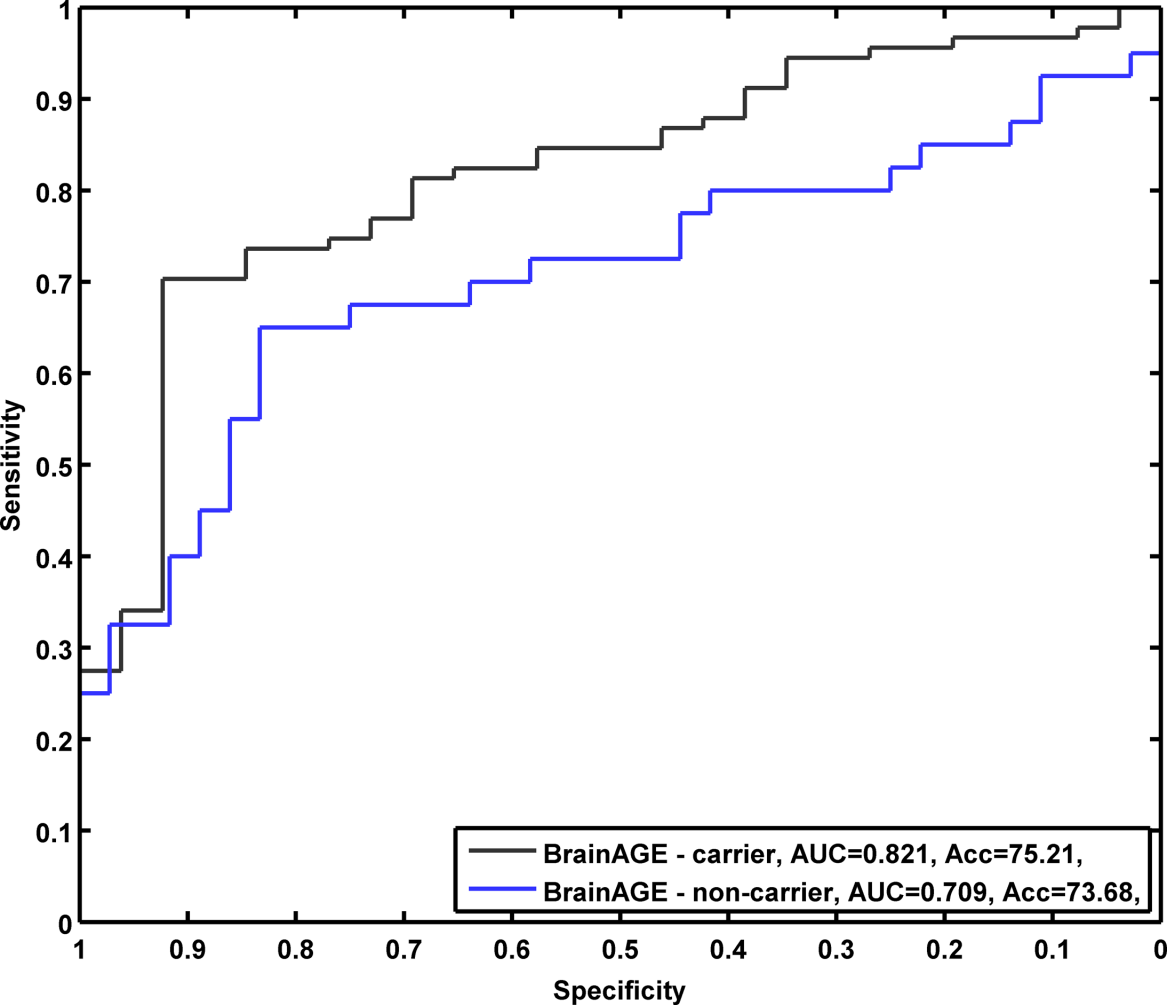
ROC curves of APOE ε4 carriers vs. non-carriers based on baseline *BrainAGE* scores in all MCI patients. Achieved accuracies (sensitivity / specificity) for predicting conversion from MCI to AD during follow-up for APOE ε4 carriers: 75% (0.70 / 0.92) and for non-carriers: 74% (0.65 / 0.83).

The effect of APOE ε4 status on prediction accuracy was further examined with ROC analyses. By varying the threshold applied to the *BrainAGE* score, ROC curves were constructed for a binary discrimination between MCI subjects who remained stable during 3 years follow-up from those who converted to AD. For the discrimination of pMCI_early from sMCI, ROC analyses at baseline *BrainAGE* scores resulted in an AUC (or c-index) of 0.88 with an accuracy rate of 85% in APOE ε4 carriers. In APOE ε4 non-carriers, prediction performances were slightly lower with an AUC of 0.75 and an accuracy rate of 78% (Fig 7). For discriminating early and late converting MCI together from sMCI patients, the AUC resulted in 0.82 for APOE ε4 carriers and 0.71 for non-carriers. Achieved accuracies for APOE ε4 carriers were 75%, for APOE ε4 non-carriers 74% (Fig 8).

Furthermore, the McNemar test was applied to explore whether predictions of future conversion to AD in MCI patients based on *BrainAGE* are significantly better than predictions based on chronological age or cognitive test scores. Predicting conversion based on baseline *BrainAGE* scores showed significantly better results as compared to chronological age and cognitive scores, especially in APOE ε4 carriers (Tables 6 and 7).

**Table 6:** Results for predicting conversion to AD in MCI subjects (APOE ε4 carriers).

	sMCI vs. pMCI^C^_early					sMCI vs. pMCI^C^ (all)				
	Accuracy (CI)	Sensitivity (CI)	Specificity (CI)	McNemar test		Accuracy (CI)	Sensitivity (CI)	Specificity (CI)	McNemar test	
				Error rate (CI)	*χ*^*2*^				Error rate (CI)	*χ*^*2*^
*BrainAGE* score*	0.85 (0.76–0.94)	0.79 (0.68–0.89)	0.92 (0.85–0.99]	0.15 (0.06–0.22)	-	0.75 (0.67–0.83]	0.70 (0.62–0.79)	0.92 (0.87–0.97)	0.25 (0.17–0.33)	-
**Chronological age**	0.39 (0.27–0.51)	0.39 (0.27–0.52)	0.92 (0.85–0.99]	0.61 (0.49–0.73)	**17.79 (*p* < 0.001)**	0.54 (0.45–0.63]	0.35 (0.26–0.44)	0.88 (0.83–0.94)	0.46 (0.37–0.55)	**7.78 (*p* < 0.01)**
**MMSE score**	0.46 (0.33–0.58)	0.52 (0.39–0.64)	0.85 (0.75–0.94]	0.54 (0.42–0.67)	**8.53 (*p* < 0.01)**	0.23 (0.15–0.31]	0.68 (0.60–0.77)	0.65 (0.57–0.74)	0.77 (0.69–0.85)	**40.01 (*p* < 0.001)**
**CDR-SB score**	0.49 (0.36–0.62)	0.70 (0.58–0.81)	0.81 (0.71–0.91]	0.51 (0.38–0.64)	**14.28 (*p* < 0.001)**	0.26 (0.18–0.34]	0.52 (0.43–0.61)	0.81 (0.74–0.88)	0.74 (0.65–0.81)	**46.12 (*p* < 0.001)**
**ADAS score**	0.69 (0.58–0.81)	0.79 (0.68–0.89)	0.69 (0.57–0.81]	0.31 (0.19–0.42)	3.20 (*n*.*s*.)	0.43 (0.34–0.52]	0.71 (0.63–0.80)	0.69 (0.61–0.78)	0.57 (0.48–0.66)	**22.44 (*p* < 0.001)**

**Bold type** = significant; **asterisk** = best performance of all models; *n*.*s*. = not significant

**Table 7:** Results for predicting conversion to AD in MCI subjects (APOE ε4 non-carriers).

	sMCI vs. pMCI^NC^_early					sMCI vs. pMCI^NC^ (all)				
	Accuracy (CI)	Sensitivity (CI)	Specificity (CI)	McNemar test		Accuracy (CI)	Sensitivity (CI)	Specificity (CI)	McNemar test	
				Error rate (CI)	χ^2^				Error rate (CI)	χ^2^
***BrainAGE* score**	**0.78 (0.68–0.89)**	**0.71 (0.59–0.82)**	**0.83 (0.74–0.93)**	**0.22 (0.11–0.32)**	**-**	**0.74 (0.64–0.84)**	**0.65 (0.54–0.76)**	**0.83 (0.75–0.92)**	**0.26 (0.16–0.36)**	**-**
**Chronological age**	0.50 (0.37–0.63)	0.83 (0.74–0.93)	0.31 (0.19–0.42)	0.50 (0.37–0.63)	8.53 (*p* < 0.01)	0.47 (0.36–0.59)	0.15 (0.07–0.23)	0.97 (0.93–1.00)	0.53 (0.41–0.64)	6.81 (*p* < 0.01)
**MMSE score**	0.60 (0.48–0.72)	0.79 (0.69–0.89)	0.58 (0.46–0.71)	0.40 (0.28–0.52)	4.17 (*p* < 0.05)	0.47 (0.36–0.59)	0.78 (0.68–0.87)	0.58 (0.47–0.69)	0.53 (0.41–0.64)	9.00 (*p* < 0.01)
**CDR-SB score**	0.67 (0.55–0.79)	0.58 (0.46–0.71)	0.75 (0.64–0.86)	0.33 (0.21–0.45)	1.64 (n.s.)	0.51 (0.40–0.63)	0.53 (0.41–0.64)	0.75 (0.65–0.85)	0.49 (0.37–0.60)	8.53 (*p* < 0.01)
**ADAS score**	0.68 (0.57–0.80)	0.92 (0.85–0.99)	0.58 (0.46–0.71)	0.32 (0.20–0.43)	0.93 (n.s.)	0.64 (0.54–0.75)	0.90 (0.83–0.97)	0.58 (0.47–0.69)	0.36 (0.25–0.46)	1.20 (*n*.*s*.)

**Bold type** = significant; *n*.*s*. = not significant

## Discussion

This study explored the effects of individual APOE ε4 status on the performance of a novel MRI-based biomarker based on the recently presented *BrainAGE* framework [15, 55] in (i) recognizing advanced brain aging in a longitudinal design and (ii) predicting prospective conversion to AD on an individual subject level.

### Longitudinal sample

AD patients as well as MCI subjects, who cognitively declined and thus converted to AD within 3 years of follow-up (pMCI), exhibited significantly larger baseline *BrainAGE* scores compared to control subjects and those with MCI, who remained cognitively stable (sMCI), but did not differ between APOE ε4 carriers and non-carriers. In contrast, brain aging accelerates more in APOE ε4 carriers during follow-up as compared to non-carriers in the pMCI and AD groups, i.e. already starting with a higher baseline *BrainAGE* score of about 6 years, brain aging accelerates during follow-up with the speed of 1.1 additional year in brain atrophy per follow-up year in pMCI ε4 carriers, but only about 0.6 years in pMCI ε4 non-carriers, and 1.7 additional years in brain atrophy per follow-up year in AD ε4 carriers and 0.9 years in AD ε4 non-carriers. This accumulated to mean *BrainAGE* scores between 7 to 9 years at the last scan, with mean follow-up durations of 2.7 years for pMCI and 1.7 years for AD. Compared to that, healthy control as well as sMCI subjects did not show any deviations from normal brain aging trajectories at baseline and follow-up.

These results are in line with recent studies that showed AD-like MRI-based indices in pMCI subjects [4, 57], increased GM atrophy of approximately 2% per year in AD [58], accelerated changes in whole brain volume in MCI [18], acceleration in atrophy rates as subjects progress from MCI to AD [59], and greater GM loss in certain regions in pMCI subjects [60, 61]. Furthermore, our results also support the assumption of AD being a form of or at least being associated with accelerated aging [18, 62, 63]. Taking into account the patients APOE genotype revealed significant differences within the MCI groups at baseline and follow-up measurements in a recent study using a MRI-based index for diagnosis and prediction of conversion [57]. In the present study, we did not find significant differences between ε4 carriers and non-carriers in baseline or follow-up *BrainAGE* scores. In contrast, ε4 carriers showed increased acceleration of individual brain aging as compared to non-carriers in pMCI and AD patients. This is in line with recent studies suggesting that APOE ε4 carriers are suffering from faster pathologic processes than non-carriers [64] and therefore have higher atrophy rates [59].

Additionally, individual *BrainAGE* scores were profoundly related to measures of clinical disease severity, most pronounced in APOE ε4 carriers and non-carriers already diagnosed with AD at baseline, as well as to measures of cognitive functioning, most pronounced in APOE ε4 carriers diagnosed with MCI at baseline and converting to AD within the next three years. Cognitive decline was recently found to progressively accelerate years before being diagnosed with AD [65], and to be correlated with the atrophy rates in specified brain regions [60]. Our results support the suggested relationship between progressive acceleration in brain aging and rate of change in cognitive functioning as well as clinical severity in pMCI and AD during follow-up, especially in APOE ε4 carriers. Furthermore, we could even show that accelerated brain aging is more closely related to the worsening of higher cognitive functions, but slightly less with disease severity in pMCI subjects, whereas in AD patients accelerated brain aging was more closely related to disease severity and slightly less with worsening of higher cognitive functions. Regarding NO and sMCI subjects a ceiling effect as well as a slightly lower variance within the cognitive scores was observed. This may be mainly due to the fact, that the scales analyzed in this study were used specifically to identify clinical disease severity as well as deterioration in cognitive functioning in the ADNI sample.

### Cross-sectional sample

Analysing the effect of APOE on the risk of conversion to AD, we compared ε4 carriers and non-carriers within the whole MCI sample. A total of 78% of ε4 carriers converted to AD within 3 years of follow-up, compared to only 53% of non-carriers, underlining a higher risk for carriers to convert to AD. Within this sample, ε4 carriers tend to convert slower than non-carriers. Several studies suggested the APOE ε4 genotype to be associated with a faster cognitive decline [66] and clinical progression of AD [30], whereas other studies doubt accelerated deterioration in APOE ε4 carriers [29, 30, 67–70]. APOE ε4 homozygosity was even suggested to slow down disease progession, since biological processes involved in AD onset and disease progression are of a different nature [29].

In our sample, having at least one ε4 allele increases time of conversion to AD by about 3 to 4 months as compared to homozygous ε3/ε3 carriers. Heterozygous ε2 carriers showed slowest conversion times. Since our sample did not include subjects with the allelic combination of ε2/ε2, we can not make any statements about average conversion times of the assumed protective allelic combination. Although ε4 carriers did not show the shortest conversion time in our study, they show the fastest brain aging, fastest cognitive decline, and highest mortality rate once converted to AD. In line with that, some studies suggest survival rates in AD patients mainly depending on their age at disease onset rather than on their APOE genotype [21, 67].

For APOE ε4 carriers as well as for non-carriers, prediction of conversion from MCI to AD was most accurate when based on baseline *BrainAGE* scores as compared to chronological age and cognitive test scores, even after inclusion of the APOE ε4 carrier-status, although prediction accuracy did not significantly improve in the *BrainAGE* prediction model. Nevertheless, prediction accuracy based on *BrainAGE* scores was higher for APOE ε4 carriers than for non-carriers, being in line with another study relating disease progression to decreases in hippocampal volume [71]. These results strongly suggest the usage of the *BrainAGE* method for screening MCI patients, aiming to find those, who are in a special high risk of conversion to AD in opposition to patients, who remain at a stable cognitive level. Identifying the quickly progressing subjects as early and secure as possible could help to prepare them best for their probable illness progression and supply them early with potential disease programs, cognitive training and medical treatments [72, 73]. Although a genetical test for APOE cannot replace any part of the clinical diagnostic procedure [35, 74, 75], including the patient’s APOE carrier status improves prediction accuracy of diagnostic tests such as memory tests or MR imaging in individuums who meet clinical MCI or AD criteria [8, 24, 76–78].

### Limitations

The present study focused on the influence of APOE status on individual brain aging trajectories in healthy subjects as well as MCI and AD patients. Therefore we divided our cohort in different APOE carrier types, based on the three allele haplotypes of the Apolipoprotein E gene, composed of ε2, ε3 and ε4. The distribution was 4% for ε2, 62% for ε3 and 34% for ε4. In caucasians, frequencies of the 3 allelic types were previously estimated 11% for ε2, 72% for ε3 and 17% for ε4 [79], respectively 8% for ε2, 77% for ε3 and 15% for ε4 [80, 81]. The underrepresentation of ε2 and ε3 and the overrepresentation of ε4 in our sample could be due to a sort of preselection in the ADNI database. Homozygous APOE ε4/ε4 carriers form about 1% to 2% of the general population [25, 34], whereas our sample included 14%. Besides, we found a relative overrepresentation of ε4 within the group of AD patients in our sample (ε4/ε4 in ADs: 21%, compared to 4% in NOs), whereas the frequency of ε2 was lower (ε2/ε3 in ADs: 3%, compared to 15% in NOs), which was also reported in several other studies [20, 21, 35]. The ADNI cohort, which was used for this study, may differ from the general population, since it only includes individuals from memory clinics, patient registries, and people recruited in public media campaigns or other forms of public advertisements. There could also exist differences in the population of North American as compared to Central Europe. This might also explain the differences in the frequencies of APOE isoforms within our sample from the estimations for the general Caucasian population.

The clinical follow-up for our ADNI cohort was done in average 1.7 years in ADs, 2.9 years in MCIs and 3.2 years in NOs. We cannot make any statement if some sMCI patients would have converted later on. Besides, group sizes of some allelic subgroups were limited to a very small number (e.g., 14 carriers and 22 non-carriers in sMCIs) due to low prevalence of some APOE isoforms or limiting selection criterions for our study. Additionally, misdiagnoses of prodromal states of AD as well as AD itself may have occured due to the possibility of mixed dementia forms or overlaying physical illness [82]. Besides, cognitive decline is a continuous process; therefore it is not always easy to securely classify the disease stage [7]. It would be of interest to repeat the study in some years in order to include longer follow-up periods and more secure diagnoses.

Furthermore, it would be interesting to examine age-specific effects of the APOE genotype on *BrainAGE* and cognitive scores, respective disease burden, as well as to verify, whether the APOE ε4 genotype is associated with an earlier age of onset, or the risk of coming down with AD, or time to conversion [26, 33, 83]. Examining age specific effects also has the potential to test, whether the correlation of APOE ε4 with the AD risk and gross brain morphology diminishes in very old age [2, 21, 84]. Aside from APOE, there are also other genetical risk factors, which probably influence MCI and AD pathogenesis. However, the inheritence of AD predisposition is very complex, with gene polymorphism and mutations interacting with each other as well as with non-genetic factors. So far, only four genes could be identifyied to influence the AD pathogenesis [7, 23, 27].

In addition, when predicting individual progression from MCI to AD, it’s impossible to take into account all possible risk factors and influencing variables like comorbidities or cognitive reserve [85]. The variability of disease progression is als reflected in the strong variations of slopes for the longitudinal *BrainAGE* changes. Generaly, abnormal brain atrophy were also found in asymptomatic subjects, whose sufficient cognitive reserve or well adapted coping methods prolongated appearance of dementia [42, 57, 85–87]. This, in turn, provokes a strong divergency between anatomical and clinical findings. But since we’ve examined flexible biological systems, we will only be able to provide estimations, but not certainty in AD diagnosis and prediction.

## Conclusions

In summary, the present study showed the potential of the *BrainAGE* method to provide more accurate results in prediciting conversion from MCI to AD than the already well-established cognitive tests, like MMSE, CDR-SB and ADAS. The knowledge of patients’ APOE genotype additionally tended to even improve prediction performance. Compared to a wide range of existing classification approaches that require disease-specific data for training, the *BrainAGE* framework uses an independent database of healthy, non-demented subjects to model the normal brain-aging pattern and consequently recognizing subtle deviations from age-related brain atrophy in new test samples. As the *BrainAGE* approach utilizes only a single T1-weighted image per subject and already has proven to work fast and fully automated with multi-centre data, it can be easily implemented in clinical routine to encourage the identification of subtly abnormal atrophy patterns as well as for monitoring treatment options.
